# Mass Spectrometry Methodology in Lipid Analysis

**DOI:** 10.3390/ijms150610492

**Published:** 2014-06-11

**Authors:** Lin Li, Juanjuan Han, Zhenpeng Wang, Jian’an Liu, Jinchao Wei, Shaoxiang Xiong, Zhenwen Zhao

**Affiliations:** Beijing National Laboratory for Molecular Sciences, Key Laboratory of Analytical Chemistry for Living Biosystems, Institute of Chemistry, Chinese Academy of Sciences, Beijing Mass Spectrum Center, Beijing 100190, China; E-Mails: linligdc@iccas.ac.cn (L.L.); hjuan@iccas.ac.an (J.H.); wang_82713@iccas.ac.cn (Z.W.); lja@iccas.ac.cn (J.L.); weijinchao@iccas.ac.cn (J.W.); mscbj@iccas.ac.cn (S.X.)

**Keywords:** lipidomics, mass spectrometry, lipids extraction, bioinformatics technology

## Abstract

Lipidomics is an emerging field, where the structures, functions and dynamic changes of lipids in cells, tissues or body fluids are investigated. Due to the vital roles of lipids in human physiological and pathological processes, lipidomics is attracting more and more attentions. However, because of the diversity and complexity of lipids, lipid analysis is still full of challenges. The recent development of methods for lipid extraction and analysis and the combination with bioinformatics technology greatly push forward the study of lipidomics. Among them, mass spectrometry (MS) is the most important technology for lipid analysis. In this review, the methodology based on MS for lipid analysis was introduced. It is believed that along with the rapid development of MS and its further applications to lipid analysis, more functional lipids will be identified as biomarkers and therapeutic targets and for the study of the mechanisms of disease.

## 1. Introduction

Lipidomics was firstly put forward in 2003 [1], in which the structures, functions and dynamic changes of lipids in cells, tissues or body fluids are investigated. Recently, it has been widely recognized that lipids are central to the regulation and control of cellular function and disease [2]. Therefore, lipidomics has gained a lot of attention and become an emerging field of basic and translational research. To date, the basic concept, research progress and potential application in drug development of lipidomics have been reviewed [3,4,5,6]. In this review, we focus on the mass spectrometry methodology for lipid analysis.

**Figure 1:**
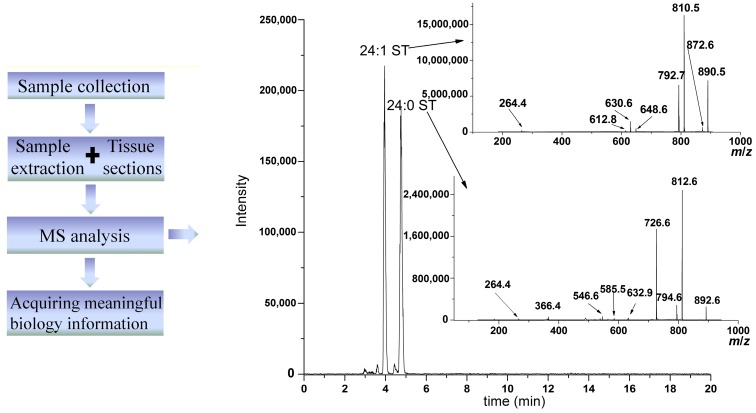
A flowchart of the study of lipidomics (ST, sulfatide).

Lipids are composed of eight categories; around 1.68 million species. The large amount of categories and the extremely complex structures of lipids lead to a formidable challenge to fully analyze all lipids. Nowadays, there are two strategies to analyze lipids: targeted lipids analysis and non-targeted lipid analysis. The targeted lipids analysis focuses on known lipids, and develops a specific method with a high sensitivity for the quantitative analysis of these specific lipids. Non-targeted lipids analysis aims to detect every lipid species simultaneously. In order to successfully realize the qualitative and quantitative analysis of lipids, many analytical methods have been developed for the analysis of lipids, including thin-layer chromatography (TLC) [7,8,9], gas chromatography (GC) [10,11,12,13], liquid chromatography (LC), enzyme-linked immunosorbent assays (ELISA) [14], nuclear magnetic resonance (NMR) [15,16] and mass spectrometry (MS) [17,18]. Among them, the MS-based method is the best in terms of high sensitivity and specificity, high throughput and high accuracy. In particular, the extensive use of electrospray ionization for lipid analysis and the improvement of mass analyzers in mass spectrometer, including the combination of different mass analyzers and the development of a high-resolution mass analyzer, has greatly increased the performance of MS in lipid analysis and revived lipid studies. In addition, the biological system is extremely complex, and it is required to extract the lipids from the biological system for further analysis. Furthermore, the studies in lipidomics have generated overwhelming amounts of data, which need bioinformatics technology to aid in data processing for acquiring meaningful biology information. Taken together, lipid analysis needs a serial of methods and technologies, including lipid extraction methods, MS-based analytical technologies and bioinformatics tools. A flowchart of the study of lipidomics is shown in Figure 1.

Herein, the methods for lipids extraction before MS detection, the ionization technologies, the mass analyzers and their applications to lipid analysis by MS are described in detail in this review. Moreover, the bioinformatics technology for data processing is also briefly discussed. New methods for lipid analysis are expected to improve the capability of lipid analysis, more functional lipids are also expected to be identified as biomarkers and therapeutic targets and for the study of the mechanisms of diseases.

## 2. The Methods for Lipid Extraction

Extracting lipids from the complex biological system is usually the first step for lipid analysis. After extraction, the proteins and some minerals are removed; therefore, the biological system becomes simple, which facilitates lipid analysis. A simple and reproducible extraction method is necessary for cross-validation of lipid data obtained in different laboratories.

So far, the most widely used extraction method was developed in the 1950s by Blight and Dyer [19], in which a mixture of methanol, chloroform and water (1:1:0.9, *v*/*v*/*v*) are used, and phase separation is involved. Lipids are dissolved in organic solvents, and proteins and other hydrophilic materials are removed after phase separation. The original Bligh and Dyer method (the BD method) is suitable for extracting major phospholipids, but not hydrophilic lipids, like lysophosphatidic acid, sphingosine-1-phosphate, sulfatide, *etc*. Modifications of the BD method have been made to increase the efficiency of extracting lipids. Among them, Yatomi *et al*. has included KCl, HCl and NH_4_OH in their extraction method to optimize extraction [20]. Milder acid, such as citric acid, has been used to replace HCl [21,22]. In addition, butanol instead of methanol and chloroform has been used in several labs as the optimized method for the extraction of lipids [21,22,23]. However, there is a concern that the acidic or alkaline conditions would induce the hydrolysis of endogenous lipids, resulting in the artificial generation of lipids [21]. For example, plasmalogens (alkenyl-acyl lipids) are extremely sensitive towards even traces of acids and produce a lysophospholipid and a fatty aldehyde. In addition, it is very hard for butanol to be evaporated, which makes the process very time-consuming. Recently, Matyash and co-workers developed a methyl-*tert*-butyl ether (MTBE)-based method to extract lipids [24], which allowed the faster and cleaner recovery of most of the major lipid classes. We also reported a methanol method, utilizing a single methanol solvent and involving only one single step of centrifugation to extract phospholipids and sphingolipids, which has been proven to be extremely simple, effective and reproducible [25]. For apolar lipids, like triacylglycerides, it was reported that the hexane-isopropanol method was best [26].

Other than the extraction method for the unbiased recovery of the lipid species mentioned above, the methods with high selectivity have been proposed for specific lipid extraction. For example, Dennis *et al*. reported that a prepurification and enrichment of free fatty acid can be achieved with the application of a bi-phasic solution of acidified methanol and isooctane [27]. In addition, a metal complex called “Phos-tag” can be used for the selective extraction of lysophosphatidic acid (LPA) and sphingosine-1-phosphate (S1P) [28]. Wenk *et al*. have selectively extracted phospho-monoester lipids by an imidazolium polymer, and after derivatization with trimethylsilyldiazomethane (TMS-diazomethane), they successfully determined the long-chain base phosphates (LCB-Ps, e.g., sphingosine-1-phosphate) [29]. Moreover, this is particularly effective for selectively extracting some lipids on the basis of their discrepancy in the adsorption capability on solid phase extraction columns through different eluents [30,31,32]. For instance, it is possible to remove those lipids with a high content using the Ostra 96 plate (Waters), such as phosphatidylcholine, lysophosphatidylcholine and sphingomyelin, while other components can be accumulated [33].

Lipid extraction should have more attention be paid to it, and before MS detection, the efficiency and reproducibility of the extraction method should be carefully tested, and the degradation and artificial generation of lipids should be avoided during the process of extraction. Furthermore, the whole process for lipid extraction should be as simple as possible to improve its operability.

## 3. The Ionization Technologies of MS

The extensive use of MS in lipids analysis is due to the development of ionization technologies. Different ionization technologies in MS were exploited for lipids analysis.

### 3.1. Electron Ionization (EI) and Chemical Ionization (CI)

**Figure 2:**
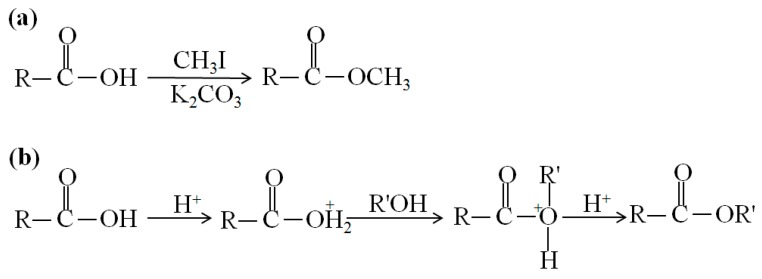
Reaction scheme for the derivatization of fatty acids. (**a**) Esterification of a fatty acid by CH_3_I and (**b**) acid-catalyzed esterification of a fatty acid.

Electron ionization (EI) is widely used in mass spectrometry, especially for the analysis of gases and volatile organic molecules, in which high energetic electrons interact with gas phase atoms or molecules to produce ions [34]. Denkert used GC-EI MS to comprehensively analyze ovarian tumor tissue’s metabolites, which showed that 51 metabolites were significantly different between borderline tumors and carcinomas tumors [35]. EI MS has been used in the determination of sterol [36], cholesterol [37] and fatty acids [38], while derivation is necessary for these nonvolatile compounds. For example, esterification is required for fatty acid analysis by GC-EI MS (Figure 2) [38,39].

The molecular ion signal in EI MS analysis is usually weak due to the high energy collision. Therefore, chemical ionization (CI) was developed to generate an easily identifiable intact molecular ion species, in which ions are produced through the collision of the analyte with ions of a reagent gas with lower energy, which are present in the ion source. Dennis’s group utilized pentafluorobenzyl bromide for derivatization of fatty acids and then employed negative chemical ionization (NCI) to successfully detect the intact signal of the molecular ion for fatty acids [27].

However, it should be noted that the EI/CI MS-based method for lipid analysis is limited, because of the unpleasant derivative steps and low sensitivity, which have restrained its further application in lipid analysis.

### 3.2. Fast Atom Bombardment (FAB)

Fast atom bombardment (FAB) has been widely used to identify the structure of nonvolatile lipids, including fatty acids [40], monoacylglycerols [41], glycerophospholipids [42,43] and sphingolipids [44,45]. However, given the complexity of lipid extracts and the inconvenience when conjugating the chromatography and FAB MS, intensive efforts need to be carried out to effectively quantitatively analyze lipids by FAB MS.

### 3.3. Matrix-Assisted Laser Desorption Ionization (MALDI)

MALDI-MS is widely used in the analyses of organic synthetic compounds, peptides and proteins for the determination of molecular ions. However, the lipid identification using MALDI-MS has been limited, due to less likely existence of a proper matrix. Although the molecular weights of the different matrices are in the range of about 150–200 g/mol, photoreactions, such as trimerizations [46] occurring upon laser irradiation, as well as incomplete matrix cluster decomposition and adduct formation, may generate a multitude of matrix peaks at higher *m*/*z* values (100–500 Da), which suppress or obscure the lipid signals with a molecular weight lower than 500 Da. In addition, the lipid extracts from a biological sample are usually a complex system, where the interferences and discriminations of different molecules make it more difficult to analyze. The choice of matrix is the most important issue for a successful MALDI-MS analysis. Among all of the matrixes, 2,5-dihydroxybenzoic acid (DHB) is predominantly used as a matrix in lipid studies. [47]. In addition, trihydroxyacetophenone (THA) [48], *p*-nitroaniline (PNA) [49], 9-aminoacridine hemihydrates (9-AA) [50] or ionic liquid matrices [51] were introduced, which demonstrated high sensitivity in the analysis of some specific lipids. Metal oxide was also chosen as a matrix for the analysis of lipid extracts from bacterial and algal sources [52], to avoid the interference of the traditional organic matrix. Recently, a report demonstrated that an aqueous suspension of citrate-capped gold nanoparticles (AuNPs) as a matrix could selectively detect triacylglycerols (TAGs) under high phosphatidylcholines (PCs) conditions [53], showing the feasibility of developing a new matrix for the selective determination of lipids.

In addition, MALDI-MS analysis has the disadvantages of rather poor reproducibility, mainly originating from the heterogeneity of the matrix-analyte crystals, which leads to MALDI-MS being heavily criticized for its quantitative analysis. A uniform matrix-analyte cocrystal minimizes the need to search for sweet spots, and more importantly, it avoids the variability of signal intensity across different locations on the target surface due to the heterogeneous crystals and greatly improves spot-to-spot reproducibility, which provides a basis for the quantitative analysis by MALDI-MS. In our group, a uniform matrix-analyte cocrystal was realized for the quantitative analysis of plasma lysophosphatidylcholines (LPCs) with the assistance of polystyrene (PS) colloidal spheres. PS spheres have superior monodispersed properties and can self-assemble to form photonic crystals. The cocrystals of the matrix and analyte deposited on the surface of photonic crystals distribute evenly, and the spot-to-spot reproducibility was satisfied with a relative standard derivation (RSD) lower than 4.1% [54].

MALDI-MS can combine with TLC and LC for lipids analysis. For example, Ida *et al.* found that the content of 10 lipids distinctly decreased in the plasma from a cystic fibrosis patient, while the content of sphingomyelin d18:0 (SM d18:0) increased by TLC-MALDI-MS analysis [55].

**Figure 3:**
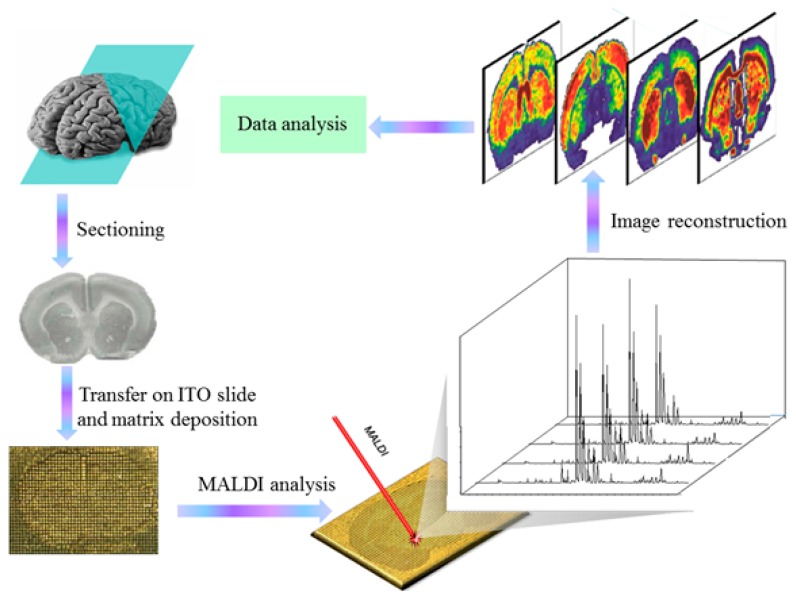
Schematic representation of the MALDI-IMS work flow.

Recent advances in MALDI-MS techniques of lipid analysis have led to the direct analysis of tissue slices with the MALDI imaging mass spectrometry (IMS) (Figure 3) [56]. The most promising advantage of MALDI-IMS is the reality to perform lipid analysis, avoiding extraction and/or separation steps, and to display the *in situ* information [57,58]. Setou *et al*. recently performed MALDI-IMS upon teeth with periodontal disease and found that an accumulation and infiltration of LPC to the root surface were related to periodontal disease [59]. High-resolution (HR) MALDI-IMS is an emerging application for the comprehensive and detailed analysis of the spatial distribution of ionized molecules *in situ* on tissue slides, and 26 molecules as highly expressed were identified in prostate carcinoma by HR MALDI-IMS [60]. Furthermore, MALDI-IMS can be even employed in single-cell lipid imaging. Römpp *et al*. combined high spatial resolution, high mass accuracy and high mass resolution MS for imaging a single Hela cell, and numerous compounds, including small metabolites, such as adenine, guanine and cholesterol, as well as different lipid classes, such as phosphatidylcholine, sphingomyelin, diglycerides and triglycerides were imaged in an individual spot of 7 μm in diameter [61].

It should be noted that the MALDI-MS analysis of lipids is still full of challenges. For example, only those rich lipids in extracts or on the tissue sections can be analyzed by MALDI-MS. It is critical to develop a new matrix for the selective determination of lipids with a low content. In addition, the stable-isotope labeling of molecules as an internal standard is an attractive technique that enables the quantitative analysis of specific molecules in a complicated system by MALDI-MS [62,63], but it is impossible to synthesize all required stable-isotope labeled compounds. The capability of quantification by MALDI-MS still needs to be improved.

### 3.4. Electrospray Ionization (ESI), Atmosphere Pressure Chemical Ionization (APCI), Atmosphere Pressure Photoionization (APPI) and Desorption Electrospray Ionization (DESI)

ESI is the major ionization method in MS for lipid analysis from body fluid, cell, bacteria, virus and tissue. “Shotgun” lipidomics was firstly proposed by Han and Gross in 2003 [1], in which ESI-MS was used for the direct analysis of lipids without pre-separation by LC. By tuning the pH value, like neutral pH in negative ion detection mode, or adding some specific ionization reagents in solution, like LiOH in positive ion detection mode, the lipids can be selectively detected [1,64]. Han *et al*. found that the sphingomyelin decreased, while ceramide increased in the brain of Alzheimer’s patients using this technology [65]. However, the phenomenon of mutual conversion and ion suppression among different lipids may lead to a systemic error when detecting complex lipid extracts by direct analysis of ESI-MS [17,66]. For example, in the ionization source, it is easy for LPC to lose the choline group and become artificial LPA, and therefore, this interferes with the measurement of LPA. Usually, to overcome these problems, a separation by liquid chromatography (LC) is needed. The HPLC separation before ESI-MS detection was established for the accurate measurement of LPA [66]. ESI is an efficient interface between LC and the mass spectrometer, which permits direct analysis of lipids as they are separated by LC, thus combining the power of LC with mass spectrometric analysis. The introduction of LC minimized the ion suppression effect. Moreover, the retention time in the LC column could also be used as another parameter for the identification of a compound other than the MS signal. For instance, Ecker *et al*. utilized the ultra performance liquid chromatography electrospray ionization tandem mass spectrometry (UPLC-ESI-SRM/MS) method to analyze the seven kinds of arachidic acids, and although some derivative molecular weights are same, they can be identified by their retention time on the column [67]. 2D-HPLC coupled to ESI was also developed to study the lipid metabolism disorder in many diseases, including obesity, hypertension, diabetes and liver cancer. Xu *et al*. used a novel on-line stop-flow 2D LC method coupled with QTOF-MS to analyze complex lipids in a plasma sample, which identified 372 lipids [68]. This group also applied on-line comprehensive silver-ion liquid chromatography (silver-ion LC) coupled with reversed-phase liquid chromatography (RPLC) to the analysis of an edible peanut oil and a mouse liver extract. As a result, 28 TAGs from the peanut oil and 44 TAGs from the mouse liver were identified [68,69].

ESI is also easily hyphenated to other separation technologies, like capillary electrophoresis [70] and a microfluidic system [71]. Because the microfluidic technique can integrate different functions on one single chip, such as the lysis of cells, the capture of lipids and the elution of captured lipids from a solid phase for the microscale purification of lipids, it may present a highly efficient technique for comprehensive lipidomics research.

Recently atmosphere pressure chemical ionization (APCI), atmosphere pressure photoionization (APPI) and desorption electrospray ionization (DESI) were also developed for lipid analysis. In APCI, the solvent acts as the chemical ionization (CI) reagent gas to ionize the samples. In APPI, a Krypton lamp producing ultraviolet light ionizes gas phase analytes. Compared with ESI, which only uses electrical fields to generate charged droplets and subsequent analyte ions by ion evaporation, APCI and APPI could provide additional mechanisms to ionize analytes. For nonpolar lipids, which cannot form charged droplets in solutions, APCI and APPI were more suitable for their analysis [69,70]. Moreover APCI and APPI are less susceptible to the effect of ionization suppression and salt buffer effects than ESI [71,72,73,74]. In 2013, Tian *et al*. compared these three ionization technologies for plasma metabolome analysis and showed that each of them has its own advantage over the other two techniques for certain types of metabolites in plasma [75]. ESI is very sensitive for detecting glycerophosphocholines, glycerophosphoethanolamines, acyl carnitines, bile acids, sulfate, *etc*. APCI is suitable for analyzing cyclic alcohols, fatty acids and linoleic acids. APPI is proven to be appropriate in detecting steroids, sphingolipids, some amino acids, nucleosides and purines in plasma [75]. DESI was first introduced by Cooks in 2005 [76], which is an ambient ionization technique, where a solvent is used for the localized extraction of molecules followed by electrospray ionization. DESI offers greater advantages with respect to clinical applications, as it can be performed under ambient conditions with minimal sample preparation, making it suitable for direct tissue analysis [77]. Hanna used DESI for the metabolic profile within lymph nodes and found that the metabolic constituent of the cancerous lymph nodes was similar to that of the primary tumor site [78].

ESI tandem MS (MS/MS) was also employed for locating double bond position in lipids. After derivatization by ozone [79,80], pyrrolidides [81], trimethylsilyloxy [82] or dimethyl disulfide [83], the derivatives yield easily recognizable key fragments, which allow for a determination of the position of the double bond. Recently, methods based on olefin cross-metathesis [84] and charge-remote fragmentation [85,86] were also proposed for the determination of double-bond positions. However, there still remains a need for simple and reliable methods to identify the double-bond positions with high accuracy and capacity for complex lipids with multi double-bonds [84].

Although ESI/APCI/APPI MS or MS/MS are extremely powerful, it is still a big challenge to identify all the lipids. Some lipids with multi-phosphate groups, like phosphoinositide, should be derived firstly to improve the sensitivity of detection [87]. Some lipids’ structures, like saccharolipids, are so complicated, that it is still a difficult task to analyze them [88]. The discrimination of isomers of lipids, like cardiolipins, is always a challenge for any MS method. In addition, the reproducibility needs to be considered for quantitative lipids analysis.

## 4. The Mass Analyzers of MS

Besides the ion source, the mass analyzer in a mass spectrometer is an extremely important part. There are many kinds of mass analyzers, including the sector magnetic analyzer, the quadrupole (Q) analyzer, the ion trap (IT) analyzer, the time of flight (TOF) analyzer, the Fourier-transform ion cyclotron resonance (FTICR) analyzer and the orbitrap analyzer. The high resolution mass analyzers, including FTICR and orbitrap, have significantly influenced the research of lipidomics, which especially facilitated direct infusion ESI MS for the simultaneous analysis of multiple lipid classes without the need for prior separation [89,90]. For example, a shotgun lipidomics approach that relies on orbitrap was established for the quantification of total lipid extracts [91]. In addition, high-resolution MS improves the confidence of molecular species assignment and the accuracy of their quantification. The fact of the below 2-ppm error in molecular weight allows the data to be retroactively searched and analyzed to characterize lipids [92].

Furthermore, with the development of tandem mass spectrometry, the analysis ability of mass spectrometry has greatly improved. QTOF-MS is usually used for non-targeted lipid analysis, which detects many metabolites simultaneously and is very helpful for drawing the metabolic disorder network. By UPLC-ESI QTOF-MS, Choi *et al*. found that the plasma lipids changed significantly after Rosuvastatin introduction, which will be helpful for understanding the side-effect mechanism caused by Rosuvastatin [93]. In addition, a triple quadrupole mass analyzer has been widely used in targeted lipids analysis. There are several detection modes in the triple quadrupole mass spectrometer, including full scan mode, single ion monitor (SIM), selected reaction monitor (SRM), multi-reaction monitor (MRM), precursor ion scan (PIS), neutral loss scan (NLS) and daughter ion scan (DIS). Using single detection mode, the triple quadrupole mass spectrometer can selectively detect one or one kind of lipid with high sensitivity and accuracy [94]. For example, by the MRM detection mode, we found that the plasma lysophosphatidylcholine (LPC) levels in colorectal cancer (CRC) patients were significantly decreased, which could be used as potential diagnostic markers for CRC disease [95]. Likewise, only some specific leukotrienes are found to be related with lung cancer by MRM detect mode analysis [96]. While taking these detection modes together, a novel multi-dimension mass spectrometry (MDMS) strategy was proposed for multiple lipids identification and quantification [97]. A full scan was firstly performed, and then DIS, PIS and NLS were executed in the MDMS strategy. MDMS can map the complete information for each individual molecular species, and therefore, it can be used to identify each molecular species in a complex lipidome.

It is believed that along with the rapid development of MS, in particular the development of mass analyzers and their further applications to lipid analysis, more functional lipids will be elucidated and identified as biomarkers and therapeutic targets.

## 5. The Bioinformatics Technology for Data Processing

The study of lipidomics, especially non-targeted lipid analysis, has generated overwhelming amounts of data, which need bioinformatics technology to aid in data processing for acquiring meaningful biology information. Data processing usually includes three parts: (1) principal component analysis (PCA) and partial least squares discriminate analysis (PLS-DA) to search for differential lipids; (2) database retrieval combined with MS/MS spectra for the identification of differential lipids; and (3) data interpretation for acquiring meaningful biology information. There exist numerous kinds of data-processing software, for example Progenesis from Waters, Clinpro from Bruker, *etc*. Some organizations, like the lipid metabolite and pathways strategy (LIPID MAPS, http://www.lipidmaps.org/resources/tutorials/bioinformaticstools.html), the human metabolome database (HMDB, http://www.hmdb.ca/spectra/spectra/ms/search), Chemspider (www.chemspider.com), *etc.*, which provide free access to their database. In addition, some websites, like www.metaboanalyst.ca, were established for aiding in data processing, free of charge. Under the assistance of analytical software, more of the functional lipidome will be identified, which will greatly enhance the understanding of the mechanisms of disease and push forward the development of lipidomics.

## 6. Outlooks and Perspectives

Lipidomics is an inter-disciplinary field in which analytical chemistry is used for the determination of the structure and content and molecule biology is used for identification of lipid function. Lipidomics is also an emerging field of basic and translational research. These years of research efforts, especially the development of the lipid metabolite and pathways strategy (LIPID MAPS), funded by NIH, have greatly pushed forward the study of lipidomics. However, lipidomics still remains at an early stage, and some issues needed to be solved. Firstly, the sample pretreatment procedures, including collection, transportation, conservation and extraction, need to be standardized; Secondly, the analytical methods and data obtained have to be cross-validated in different laboratories; Thirdly, analytical approaches for the accurate analysis of some lipids, such as gangliosides, phosphoinositides, pregnenolone, *etc.*, are still lacking; Fourthly, still less attention has been paid to improving the data interpretation, and the informatics technologies are urgently expected to be improved for acquiring meaningful biology information from lipid data.
